# Supplementary material for: Terrestrial-type nitrogen-fixing symbiosis between seagrass and a marine bacterium
Source: Nature. 2021 Nov 3;600(7887):105–9. doi: 10.1038/s41586-021-04063-4 (PMC8636270; doi:10.1038/s41586-021-04063-4)
Supplement: Supplementary file 1 — Supplementary Notes 1–11. [file 41586_2021_4063_MOESM1_ESM.pdf]

nature portfolio

https://doi.org/10.1038/s41586-021-04063-4

Supplementary information

Terrestrial-type nitrogen-fixing symbiosis between seagrass and a marine bacterium

In the format provided by the authors and unedited

Supplementary Information

Terrestrial-type N-fixing symbiosis between seagrass and a marine bacterium

Authors: Wiebke Mohr[1*], Nadine Lehnen[1], Soeren Ahmerkamp[1], Hannah K. Marchant[1], Jon S. Graf[1], Bernhard Tschitschko[1], Pelin Yilmaz[1†], Sten Littmann[1], Harald Gruber-Vodicka[1], Nikolaus Leisch[1], Miriam Weber[2], Christian Lott[2], Carsten J. Schubert[3], Jana Milucka[1], Marcel M. M. Kuypers[1]

Affiliations:
[1]Max Planck Institute for Marine Microbiology, Celsiusstraße 1, 28359 Bremen, Germany
[2]HYDRA Marine Sciences GmbH, Steinfeldweg 15, 77815 Bühl, Germany
[3]Swiss Federal Institute of Aquatic Science and Technology (Eawag), Seestraße 79, 6047 Kastanienbaum, Switzerland
[†] Present address: Institute for Artificial Intelligence in Medicine, University Hospital Essen, Data Science Research Group, Girardetstraße 2, 45131 Essen, Germany

[*] For correspondence: Wiebke Mohr, Max Planck Institute for Marine Microbiology, Celsiusstraße 1, 28359 Bremen, Germany, wmohr@mpi-bremen.de

Supplementary Notes included:

1) Primary production supported by root-associated N_2 fixation
2) Calculating the transfer of freshly fixed N from $^{15}N_2$ fixation by *C. neptuna* to root biomass
3) Comparison of nanoSIMS-based mass-balanced ^{15}N root enrichments to bulk root biomass measurements
4) Ammonium transfer from *C. neptuna* to *P. oceanica*
5) Amino acid transfer from *C. neptuna* to *P. oceanica*
6) Average protein $\delta^{15}N$ based on amino acid concentration and isotopic composition
7) New species *Celerinatantimonas neptuna* - Whole genome comparison of *C. neptuna* and *C. diazotrophica*
8) Additional metabolic traits for interactions with seagrass
9) Proposed reclassification of the genera *Celerinatantimonas, Agarivorans, Aliagarivorans* and *Alginatibacterium* into the family *Celerinatantimonadaceae*
10) Root microbiome of *P. oceanica*
11) List of genes indicated in Fig. 4a and Extended Data Fig. 6

1) Primary production supported by root-associated N_2 fixation

The amount of primary production that can be sustained by root-associated N_2 fixation was calculated using the following steps:

1) For roots, rhizomes and leaves, the total amount of N fixed (roots and rhizomes) or transferred (leaves) per day and tissue (N_{tissue} in µmol N DW_{tissue}^{-1} d^{-1}) was calculated as follows:

$$N_{tissue} = NF_{tissue} \times DW_{tissue} \quad \text{(Equation 1)}$$

with NF_{tissue} = tissue-specific N_2 fixation (roots and rhizomes) or N transfer (leaves) in µmol N g_{DW}^{-1} d^{-1} and DW_{tissue} = total amount of tissue dry weight (in g $shoot^{-1}$). Dry weights were measured from plants sampled in August 2016 (an N_2 fixation season) with 2.06, 10.84 and 1.98 g $shoot^{-1}$ for leaves, rhizomes and roots, respectively.

2) For each tissue, the amount of carbon biomass that can be supported by the amount of fixed N (C_{tissue} in µmol C g DW_{tissue}^{-1} d^{-1}) was calculated as follows:

$$C_{tissue} = N_{tissue} \times \text{molar C:N ratio}_{tissue} \quad \text{(Equation 2)}$$

with molar C:N $ratio_{tissue}$ = tissue-specific molar carbon to nitrogen ration obtained through the elemental analyzer (average C:N ratios of leaves: 46, rhizomes: 94, roots: 85). C_{tissue} was 53.5-72.2, 0.0-17.4 and 0.7-8.9 µmol C g DW_{tissue}^{-1} d^{-1} for roots, rhizomes and leaves, respectively.

3) The amount of daily primary production (PP in mmol C m^{-2} d^{-1}) that can be supported by plant-associated N_2 fixation was then calculated as follows:

$$C_{shoot}\ (\mu\text{mol C shoot}^{-1}\ d^{-1}) = C_{roots} + C_{rhizomes} + C_{leaves} \quad \text{(Equation 3)}$$

and

$$PP = C_{shoot} \times \text{shoots m}^{-2} / 1000 \quad \text{(Equation 4)}$$

with shoots m^{-2} = shoot density per meter square measured in June 2019 (607 shoots m^{-2}).

The amount of daily primary production (PP) that can could be sustained by N_2 fixation activity was 44-49 mmol C m^{-2} d^{-1} matching well with the net CO_2 flux of ~ 50 mmol CO_2 m^{-2} d^{-1} measured using the eddy covariance technique.

2) Calculating the transfer of freshly fixed N from $^{15}N_2$ fixation by *C. neptuna* to root biomass

A total of 167 nanoSIMS images were obtained from two sections prepared from N_2-fixing plants, one in each June 2014 and August 2016. Images were obtained from all tissue types across the root section (from rhizoplane to stele), and the combined data set is therefore considered representative of the root material. NanoSIMS-based ^{15}N-enrichment in single cells and root tissue were combined with their areal extent (through image analysis, see methods) to obtain mass-balanced ^{15}N enrichments of the total root biomass (plant and bacteria; AP_{total}) according to:

$$AP_{symbiont} \text{ x } area_{symbiont} + AP_{host} \text{ x } area_{host} = AP_{total} \text{ x } area_{total} \quad \text{(Equation 5)}$$

with $AP_{symbiont}$ and AP_{host} the atom percent (at%) ^{15}N excess in the *C. neptuna* symbiont cells (June: 0.02217 (0.01850) at% and August: 1.55396 (1.22214) at%; mean (median)), the host root tissue (June: 0.00889 at%, and August: 0.01318 at%), respectively, and with $area_{symbiont}$, $area_{host}$ and $area_{total}$ the surface area occupied by *C. neptuna* symbiont cells (June: 0.000156 mm^2, August: 0.000134 mm^2), host root tissue (June: 0.260 mm^2, August: 0.0397 mm^2) and the sum of these two areas, respectively. The mass-balanced total biomass yielded for June: 0.00941 (0.00835) at% and for August: 0.35433 (0.04050) at%; mean (median)). Differences in nitrogen content between bacterial cells (assumed C:N ratio of 5) and the root tissue (average C:N ratio of 85 as measured through EA-IRMS) were taken into account when using the area as a proxy for the biomass. The at% ^{15}N excess above was calculated as the mean (and median) ^{15}N enrichment in *C. neptuna* cells or root tissue minus the at% ^{15}N natural abundance (0.36719 at% and 0.36747 at% for June and August, respectively) measured in the bulk root biomass of the control incubations (without added $^{15}N_2$) or time-zero samples (see methods).
Solving Equation 5 for either symbiont or host, the percentage (based on mean (median) of ^{15}N excess) of freshly fixed N that remained within the symbiont cells or was transferred to the root tissue was calculated:

June 2014
Remained in symbiont: 2 (2) %
Transfer to root: 98 (98) %,
August 2016
Remained in symbiont: 20 (63) %
Transfer to root: 80 (37) %

3) Comparison of nanoSIMS-based mass-balanced ^{15}N root enrichments to bulk root biomass measurements

The mass-balanced ^{15}N enrichments of total root biomass compared quite well with the bulk biomass measurements:

For June 2014:
NanoSIMS-based mass-balanced ^{15}N enrichment of total root biomass:
0.37555 at% ^{15}N (median-based) and **0.37661 at% ^{15}N** (mean-based)
Bulk root biomass ^{15}N enrichments of the respective plant:
0.3885 at% ^{15}N (median), **0.38574 at% ^{15}N** (mean), **0.367809 – 0.398145 at% ^{15}N** (range)

For August 2016:
NanoSIMS-based mass-balanced ^{15}N enrichment of total root biomass:
0.40868 at% ^{15}N (median-based) and **0.72252 at% ^{15}N** (mean-based)
Bulk root biomass ^{15}N enrichments of the respective plant:
0.38784 at% ^{15}N (median), **0.39442 at% ^{15}N** (mean), **0.373661 – 0.448952 at% ^{15}N** (range)

The mass-balanced ^{15}N enrichments do not exactly match ^{15}N measurements of bulk root biomass (using EA-IRMS; see methods), but the values fall within the measured range. The difference is likely due to the relatively large variability of N_2 fixation rates associated with individual root pieces (see Fig. 1), and different root pieces are used for the different analyses.

4) Ammonium transfer from *C. neptuna* to *P. oceanica*

The primary product of N_2 fixation, ammonium, was likely transferred from the N_2-fixing *C. neptuna* to its host *P. oceanica*. However, the gene expression of the glutamate dehydrogenase (*gdhB*) by *C. neptuna* indicates that ammonium produced from N_2 fixation was, at least partially, incorporated into the amino acid glutamate. The subsequent synthesis of glutamine via glutamine synthetase (*glnA),* typically used for bacterial biomass production, was likely inhibited though, judging from the high expression levels of the GlnA-inhibiting *glnB*-type P_{II} protein[96,97] (Extended Data Fig. 6 and 7). A similar downregulation/inhibition of bacterial glutamine synthesis occurs in terrestrial Rhizobia-legume symbioses where it acts to prevent Rhizobia from assimilating ammonium, causing a release to the plant[20,98]. To facilitate the N release and avoid N limitation by the rhizobia, the host plant provides an amino acid to the rhizobia[27-29]. Based on the transcriptome data, *P. oceanica* might provide *C. neptuna* with the amino acid GABA in return for ammonium and/or other amino acids (Extended Data Fig. 9), analogous to some Rhizobia-legume symbioses[28,29].

5) Amino acid transfer from *C. neptuna* to *P. oceanica*

The use of the ^{15}N tracer in our incubations allowed determining the N source and, with that, the primary location of synthesis (bacteria or plant) for the different amino acids. Due to the enrichment of ^{15}N in the substrate (N_2 gas), any ^{15}N enrichment (above natural abundance) in the amino acids can only originate from N_2 fixation. N_2 fixation is the most important 'new' N source for the seagrass in summer, however, recycled N sources are also used in plant metabolism, and these unlabeled N sources dilute the ^{15}N signal in the amino acids[99,100].

The primary product of the incorporation of ammonium produced upon N_2 fixation is the amino acid glutamate (from the incorporation of ammonium into 2-oxoglutarate (2-OG)). The only other amino acid that is generally synthesized through the direct incorporation of ammonium is glutamine (from glutamate). Most other amino acids are usually synthesized through transamination of carbon backbones or other amino acids (or intermediates) with glutamate.

In order to distinguish the amino acids synthesized by the N_2-fixing microorganism from those synthesized by the plant upon transfer of freshly fixed N, we compared the ^{15}N enrichment of individual amino acids to the ^{15}N enrichment of glutamate in the root tissue.

Glutamate had the highest ^{15}N enrichment of all measured amino acids, confirming the primary incorporation of freshly fixed, ^{15}N-labeled N. If the synthesized, ^{15}N-enriched glutamate would be the only amino acid transferred from the bacteria to the plant, any downstream synthesis of other amino acids by the plant would yield ^{15}N-enrichments lower than those of glutamate due to the dilution with unlabeled glutamate in the plant metabolism. However, two other amino acids, phenylalanine and leucine, also had ^{15}N enrichments very close to that of glutamate (Extended Data Fig. 8) indicating that these two amino acids are also synthesized by the N_2-fixing microorganism rather than by the plant. All other amino acids had lower ^{15}N enrichments that differed between the various amino acids (Extended Data Fig. 8) suggesting that they were primarily synthesized by the plant upon receiving glutamate, phenylalanine and/or leucine from the bacteria.

In addition to the amino acids glutamate, phenylalanine and leucine, ammonium formed upon N_2 fixation may also have leaked out of the bacterial cells into the root tissue and was subsequently incorporated by the plant (see above). The leakage of ^{15}N-labeled ammonium alone, though, cannot be the only route of freshly fixed N supply from the bacteria to the plant. The ^{15}N enrichment of the amino acids would be rather low and relatively uniform across all amino acids due to the continuous dilution of the ^{15}N signal with recycled, unlabeled ammonium in the plant metabolism. It is likely, however, that ^{15}N-labeled ammonium contributed to the fixed N supply from the bacteria to the plant similar to observations of rhizobia-legume symbioses[101].

Generally, amino acids that are synthesized with carbon backbones of the glycolysis and pentose phosphate pathway (PPP) had higher ^{15}N enrichments than those amino acids synthesized with carbon backbones of the TCA cycle with the exception of alanine and lysine (Extended Data Fig. 8). Alanine had the lowest ^{15}N enrichment relative to glutamate. Interestingly, alanine is a primary product of rhizobial N_2 fixation and is believed to be the amino acid transferred to the legume host[102]. Lysine contains two nitrogen atoms and is synthesized through transamination of aspartate with glutamate. Since aspartate was not as highly enriched (Extended Data Fig. 8), the high enrichment of ^{15}N in lysine primarily originates from the second transamination step rather than from the prior synthesis of aspartate.

Together these results indicate that, besides ammonium, the amino acids glutamate, phenylalanine and leucine are the primary products of N_2 fixation by *C. neptuna* and are transferred to *P. oceanica* (Extended Data Fig. 9).

6) Average protein $\delta^{15}N$ based on amino acid concentration and isotopic composition

The ^{15}N enrichments in individual amino acids were used in combination with their concentrations (GC with FID) to obtain an amino acid-based mass balance and an average protein ^{15}N enrichment. Since cellular protein often constitutes a majority of biomass, the average protein ^{15}N enrichment was then compared to bulk biomass ^{15}N enrichments.

For two of the analyzed plants, eleven amino acids were quantifiable using GC-FID and the $\delta^{15}N$ was retrievable using GC-IRMS. These two plants were used for mass balancing the average protein ^{15}N enrichment according to:

$$\text{average protein } \delta^{15}N = \sum (N_{AA} / N_{total\text{-}AA} * \delta^{15}N_{AA}) \quad \text{(Equation 6)}$$

with $N_{AA} / N_{total\text{-}AA}$ equaling the fraction of N in each of the eleven amino acids over total N (sum of N of all eleven amino acids) such that the sum of the fractions = 1, and the $\delta^{15}N_{AA}$ equaling the ^{15}N enrichment in each of the individual amino acids.

The eleven amino acids that were mass balanced were Alanine, Aspartate, Glutamate, Glycine, Leucine, Lysine, Phenylalanine, Proline, Serine, Threonine, and Valine.

The mass-balance yielded average protein $\delta^{15}N$ for the two plants of 31 ‰ and 87 ‰, which compare to averages of 173 ‰ and 77 ‰, respectively, from bulk measurements of these two plants. Although cellular protein usually constitutes a major portion of cellular N, deviations between the average protein $\delta^{15}N$ and the average $\delta^{15}N$ of the bulk root biomass can be caused

by, for example, non-protein N such as ^{15}N-enriched ammonium from N_2 fixation that has not been incorporated yet. In addition, not all amino acids were measured here, and those could potentially also contribute to the higher difference observed for one plant here.

7) New species *Celerinatantimonas neptuna* - Whole genome comparison of *C. neptuna* and *C. diazotrophica*

The 16S rRNA gene sequences in our metagenome-assembled genome (MAG) of the N_2-fixing symbiont in the roots of *P. oceanica* had a similarity of ~95% with those of *Celerinatantimonas diazotrophica*[25]. Based on species and genus discernment[26], this symbiont represents a new species within the genus *Celerinatantimonas*, which we named *Celerinatantimonas neptuna* (Fig. 2c).

The MAGs of *C. neptuna* and its closest cultured relative *C. diazotrophica* (DSM18577) were of similar size and GC content and shared 2422 protein-coding genes (~65% of protein coding genes) with overall low synteny (Extended Data Fig. 6). Both genomes encoded six rRNA operons, and multiple rRNA operons are common in gammaproteobacteria[103].

Among genomic regions that were exclusively present in *C. neptuna*, two regions encoded for the synthesis and transport of hemagglutinin, one region encoded for the synthesis of lipopolysaccharides (LPS), and seven regions were predicted secondary metabolite clusters (Extended Data Fig. 6). Hemagglutinins, also known as lectins, are large, glycan-binding proteins secreted to the cell surface and act as recognition molecules[104,105]. While plant-derived lectins are usually associated with defense mechanisms or symbioses with N_2-fixing rhizobia, bacteria-derived lectins are commonly associated with pathogenicity[104]. However, genes coding for hemagglutinin have also been found in the plant growth-promoting endophyte *Enterobacter* sp. 638 (ref. [106]). Lipopolysaccharides are cell surface components that play a vital role in pathogenic and beneficial plant-microbe interactions (e.g. ref. [107]). In symbioses of N_2-fixing bacteria with plants, LPS may play a role in, for example, adherence, virulence, nodule formation and/or nodule maintenance[108]. From the seven secondary metabolite clusters, five were predicted non-ribosomal polypeptide (NRPS) or polyketide synthases (PKS). The NRP(K)S as well as the other two secondary metabolite clusters of *C. neptuna* were predicted to synthesize antibiotic/antimicrobial compounds and siderophores which may play an important role in suppressing growth of competitors and promoting plant growth and health[35,37,109]. These genomic differences between *C. neptuna* and C. *diazotrophica* may indicate host-specific adaptations in *C. neptuna*.

8) Additional metabolic traits for interactions with seagrass

In addition to the gene regions encoding for lipopolysaccharides and hemagglutinin described above, the genome of *C. neptuna* also contained genes encoding for chemotaxis (*che* genes), attachment to plant cells (tight adhesion; TAD), plant cell wall degradation (*celZ*, *bglB* (cellulose) and *pemA*, *pelB* (pectin)), the synthesis of growth promoting secondary metabolites (indole-3-acetic acid, acetoin and 2,3-butanediol) and effector secretion (secretion systems: TAT, SEC, T1SS, T2SS, T4SS). Many of these traits are considered beneficial for the interaction with plants and commonly occur in endophytes[35]. The high transcription of a phenolic acid decarboxylase (*padC*; Extended Data Fig. 6) indicates that *C. neptuna*, just like members of the seagrass rhizosphere[110], degrades antimicrobial phenolic acids produced by the seagrass.

9) Reclassification of the genera *Celerinatantimonas*, *Agarivorans*, *Aliagarivorans* and *Alginatibacterium* from family *Psychromonadaceae* to family *Celerinatantimonadaceae*

The genera *Celerinatantimonas*, *Agarivorans*, *Alginatibacterium* and *Aliagarivorans* closely cluster together in our phylogenetic tree based on the 16S rRNA gene. The similarity of the 16S rRNA genes between members of the genus *Celerinatantimonas* and the other three genera is ≥89.8%. Based on this clustering and the 16S rRNA gene similarity[26], we propose to include the four genera *Celerinatantimonas*, *Agarivorans*, *Alginatibacterium* and *Aliagarivorans*, currently in the polyphyletic *Psychromonadaceae*, into one family, the *Celerinatantimonadaceae*. This classification is also supported by the clustering of whole genomes in the Genome Taxonomy Database (GTDB[111]).

Emended description for the family Celerinatantimonadaceae: Current known members of the *Celerinatantimonadaceae* are characterized by the capacity to fix N_2 (Suppl. File 3).

10) Root microbiome of *P. oceanica*

The root-associated microbial communities of individual *P. oceanica* plants were dominated by only eleven bacterial classes, which had relative abundances of the 16S rRNA gene reads above 1.5%: Acidimicrobiia, Alphaproteobacteria, Anaerolineae, Bacteroidia, Campylobacteria, Clostridia, Deltaproteobacteria, Fusobacteria, Gammaproteobacteria, Planctomycetacia, and Spirochaeta (Extended Data Fig. 3). Root-associated bacterial communities, however, were quite variable between individual plants (Extended Data Fig. 3). Furthermore, the root microbiomes of non-N_2-fixing plants were significantly different from those of N_2-fixing plants with differences largely driven by a single OTU, identified as *C. neptuna* (Extended Data Fig. 3).

The importance of certain microorganisms and/or their functional traits is commonly assessed by determining the core microbiome, i.e. those OTUs occurring across many individuals or samples. The assessments use various stringencies, which range from an OTU occurring in 30% up to 100% of subjects (for review see ref. [112]). We therefore assessed the shared root microbiome at different levels of stringency (Extended Data Fig. 3). Initially, we identified OTUs that occurred in at least one plant in both the N_2-fixing and non-N_2-fixing categories. Subsequently we looked for OTUs that were present in at least 10% of the plants in the N_2-fixing category and at least 10 % of the plants in the non-N_2-fixing category, then 20% of plants and so on up to 100%, in steps of 10%. The number of shared OTUs decreased exponentially with increasing stringency (Extended Data Fig. 3). While 2018 of 13886 OTUs were present in at least one plant of both categories, only 51 OTUs (0.37%) were present in at least half of the non-N_2-fixing and the N_2-fixing plants. Of these 51 OTUS, representing ten of the eleven major bacterial classes, one third belonged to the Alphaproteobacteria (17) followed by the Gammaproteobacteria (13), Deltaproteobacteria (7) and Acidimicrobiia (4). The classes Bacteroidia, Anaerolineae, Campylobacteria and Clostridia each were represented by two OTUs while the classes Fusobacteria and Spirochaeta were represented by one OTU each. Several of these OTUs were closely related to known bacteria that have the potential to fix N_2 (e.g. *Celerinatantimonas*, *Vibrio*, *Tistlia*), reduce sulfate (e.g. *Desulfatitalea*, *Desulfotalea*, *Desulfatiglans*), or oxidize sulfide (e.g. *Arcobacter, Thiodiazotropha*). The beneficial effects of N_2 fixation and sulfide oxidation to the plant seem obvious: a source of N at times of N limitation and the detoxification of harmful H_2S. Sulfide oxidizers are among the most commonly found microorganisms associated with the roots of aquatic plants[113] and likely protect the plant from the detrimental effect of H_2S (e.g. ref. [114]). Sulfide may be toxic to the plant, but can be detoxified either by radial oxygen loss from the roots, by sulfur incorporation into root tissue or by sulfide oxidizers associated with the plant or the adjacent sediments[115]. The potential benefit of sulfate-reducing bacteria, which produce H_2S, is less straightforward. Nonetheless, it has been suggested that sulfate-reducing bacteria may also be beneficial to seagrasses[116]. Microorganisms involved in the sulfur cycle as well as N_2 fixation are frequently found in seagrass root microbiomes[24,117,118].

11) List of genes indicated in Fig. 4a and Extended Data Fig. 6

Phage_1-2 (prophages), RNF1 (Rhodobacter Nitrogen Fixation complex 1), Sec_meta_1-7 (secondary metabolite gene clusters), rRNA_c1-6 (rRNA gene clusters), hemaglut_1-2 (hemagglutinin gene clusters), LPS/surface (lipopolysaccharide and cell surface gene cluster), *nqrA* (Na(+)-translocating NADH-quinone reductase subunit A), hypoth. (hypothetical), *gapA_1* (Glyceraldehyde-3-phosphate dehydrogenase A), *flaA* (flagellin), Protein_synthesis_1 (Elongation factor Ts, 30S ribosomal protein S2), *gabT* (Gamma-aminobutyrate:alpha-ketoglutarate or similar aminotransferase), *lpxC* (UDP-3-O-acyl-N-acetylglucosamine deacetylase), *groL/groS* (chaperonin), *dusB* (tRNA-dihydrouridine synthase B), *fis* (DNA-binding protein Fis), *hupA* (DNA-binding protein HU-alpha), *azoR* (FMN-dependent NADH-

azoreductase), Protein_synthesis_1 (ribosomal proteins, RNA polymerase, *secY*), Protein_synthesis_2 (ribosomal proteins), Protein_synthesis_3 (ribosomal proteins), *ahpC/F* (Alkyl hydroperoxide reductase protein subunit C and F), Protein_synthesis_4 (elongation factors, ribosomal proteins), Protein_synthesis_5 (RNA polymerase, ribosomal protein), *tufB_2* (Elongation factor Tu 2), Protein_synthesis_6 (ribosomal proteins), *rebB* (killing trait protein repB), *ydfZ* (Putative selenoprotein YdfZ), *glnA* (Glutamine synthetase), *copA* (Copper-exporting P-type ATPase), *hsp* (small heat shock protein), *pstS* (Phosphate-binding protein gene PstS), *gabT* (Gamma-aminobutyrate:alpha-ketoglutarate or similar aminotransferase), ATP_synthase (ATP synthase subunits), *dps* (dna protecting protein under starved conditions; ferritin-like), Protein_synthesis_7 (ribosomal proteins), *ilvC* (Ketol-acid reductoisomerase ($NADP^+$)), *adh* (Alcohol dehydrogenase), omp (outer mebrane protein), *luxR* (HTH-type transcriptional regulator LuxR), *pgk/fbaA* (Phosphoglycerate kinase and Fructose-bisphosphate aldolase class 2), Protein_synthesis_8 (ribosome maturation factor Rim, tRNA methyltransferase, ribosomal protein), rpsT (30S ribosomal protein S20), P-II (Nitrogen regulatory protein P-II), Protein_synthesis_9 (ribosomal and primosomal proteins), *rpoD* (RNA polymerase sigma factor RpoD), *rpsU* (30S ribosomal protein S21), *padC* (Phenolic acid decarboxylase PadC), *eno_1* (Enolase), *pykF* (Pyruvate kinase I), *grcA* (stress-induced alternate pyruvate formate-lyase subunit), *modE* (molybdenum-dependent transcriptional regulator), *eno_2* (Enolase), *dnaK/J* (Chaperone proteins DnaK/J), *ftsH* (ATP-dependent zinc metalloprotease FtsH), *infB* (Translation initiation factor IF-2), PTS (PTS system glucose-specific EIIA component; Phosphoenolpyruvate-protein phosphotransferase; Phosphocarrier protein HPr), *cysK* (Cysteine synthase A), *nifF* (Flavodoxin 2), *sacB* (Levansucrase), *nifHDK* (nitrogenase gene cluster), *lon_1* (Lon protease), *cspC* (Cold shock-like protein CspC), *cspD* (Cold shock-like protein CspD), *htpG* (Chaperone protein HtpG), *ompC* (Outer membrane porin C), *cydA/B* (Cytochrome bd-I ubiquinol oxidase subunit 2/Cytochrome bd ubiquinol oxidase subunit 1), *ihfA* (Integration host factor subunit alpha), Protein_synthesis_10 (50S ribosomal protein L20, 50S ribosomal protein L35, Translation initiation factor IF-3), Protein_synthesis_11 (50S ribosomal protein L32, Large ribosomal RNA subunit accumulation protein YceD), *tpiA* (Triosephosphate isomerase), hypoth./*ycjG_2* (hypothetical protein; L-Ala-D/L-Glu epimerase), *flp* (pilus assembly protein flp), *ihfB* (Integration host factor subunit beta), *rpsA* (30S ribosomal protein S1), *cspC* (Cold shock protein-like CspC), *pflB* (Formate acetyltransferase 1).

Supplementary References

96 Arcondéguy, T., Jack, R. & Merrick, M. P-II signal transduction proteins, pivotal players in microbial nitrogen control. *Microbiology and Molecular Biology Reviews* **65**, 80-105, doi:10.1128/mmbr.65.1.80-105.2001 (2001).

97 Forchhammer, K. P-II signal transducers: novel functional and structural insights. *Trends in Microbiology* **16**, 65-72, doi:10.1016/j.tim.2007.11.004 (2008).

98 Patriarca, E. J., Tatè, R. & Iaccarino, M. Key role of bacterial NH_4^+ metabolism in rhizobium-plant symbiosis. *Microbiology and Molecular Biology Reviews* **66**, 203-+, doi:10.1128/mmbr.66.2.203-222.2002 (2002).
99 Knowles, T. D. J., Chadwick, D. R., Bol, R. & Evershed, R. P. Tracing the rate and extent of N and C flow from ^{13}C,^{15}N-glycine and glutamate into individual de novo synthesised soil amino acids. *Organic Geochemistry* **41**, 1259-1268, doi:10.1016/j.orggeochem.2010.09.003 (2010).
100 Charteris, A. F. ^{15}N Tracing of Microbial Assimilation, Partitioning and Transport of Fertilisers in Grassland Soils. *Springer Theses-Recognizing Outstanding PhD Research*. Springer Nature, Cham (2019).
101 Li, Y. Z., Parsons, R., Day, D. A. & Bergersen, F. J. Reassessment of major products of N_2 fixation by bacteroids from soybean root nodules. *Microbiology-SGM* **148**, 1959-1966, doi:10.1099/00221287-148-6-1959 (2002).
102 Waters, J. K. *et al.* Alanine, not ammonia, is excreted from N_2-fixing soybean nodule bacteroids. *Proceedings of the National Academy of Sciences of the United States of America* **95**, 12038-12042, doi:10.1073/pnas.95.20.12038 (1998).
103 Větrovský, T. & Baldrian, P. The variability of the 16S rRNA gene in bacterial genomes and its consequences for bacterial community analyses. *PloS One* **8**, doi:10.1371/journal.pone.0057923 (2013).
104 Sharon, N. & Lis, H. History of lectins: from hemagglutinins to biological recognition molecules. *Glycobiology* **14**, 53R-62R, doi:10.1093/glycob/cwh122 (2004).
105 Nizet, V., Varki, A. & Aebi, M. Microbial lectins: hemagglutinins, adhesins, and toxins. (2017).
106 Taghavi, S. *et al.* Genome sequence of the plant growth promoting endophytic bacterium *Enterobacter* sp. 638. *PloS Genetics* **6**, doi:10.1371/journal.pgen.1000943 (2010).
107 Newman, M.-A., Dow, J. M., Molinaro, A. & Parrilli, M. Invited review: priming, induction and modulation of plant defence responses by bacterial lipopolysaccharides. *Journal of Endotoxin Research* **13**, 69-84 (2007).
108 Serrato, R. V. Lipopolysaccharides in diazotrophic bacteria. *Frontiers in Cellular and Infection Microbiology* **4**, doi:10.3389/fcimb.2014.00119 (2014).
109 Gaiero, J. R. *et al.* Inside the root microbiome: Bacterial root endophytes and plant growth promotion. *American Journal of Botany* **100**, 1738-1750, doi:10.3732/ajb.1200572 (2013).
110 Sogin, E. M. *et al.* Sugars dominate the seagrass rhizosphere. *bioRxiv*, 797522 (2021).
111 Parks, D. H. *et al.* A standardized bacterial taxonomy based on genome phylogeny substantially revises the tree of life. *Nature Biotechnology* **36**, 996-1004 (2018).
112 Hernandez-Agreda, A., Gates, R. D. & Ainsworth, T. D. Defining the Core Microbiome in Corals' Microbial Soup. *Trends in Microbiology* **25**, 125-140, doi:10.1016/j.tim.2016.11.003 (2017).
113 Küsel, K., Trinkwalter, T., Drake, H. L. & Devereux, R. Comparative evaluation of anaerobic bacterial communities associated with roots of submerged macrophytes growing in marine or brackish water sediments. *Journal of Experimental Marine Biology and Ecology* **337**, 49-58, doi:10.1016/j.jembe.2006.06.004 (2006).

114 Martin, B. C. *et al.* Oxygen loss from seagrass roots coincides with colonisation of sulphide-oxidising cable bacteria and reduces sulphide stress. *The ISME Journal* **13**, 707-719, doi:10.1038/s41396-018-0308-5 (2019).
115 Lamers, L. P. M. *et al.* Sulfide as a soil phytotoxin-a review. *Frontiers in Plant Science* **4**, doi:10.3389/fpls.2013.00268 (2013).
116 Brodersen, K. E. *et al.* Seagrass-mediated phosphorus and iron solubilization in tropical sediments. *Environmental Science & Technology* **51**, 14155-14163, doi:10.1021/acs.est.7b03878 (2017).
117 Crump, B. C., Wojahn, J. M., Tomas, F. & Mueller, R. S. Metatranscriptomics and Amplicon Sequencing Reveal Mutualisms in Seagrass Microbiomes. *Frontiers in Microbiology* **9**, doi:10.3389/fmicb.2018.00388 (2018).
118 Cúcio, C., Engelen, A. H., Costa, R. & Muyzer, G. Rhizosphere microbiomes of European seagrasses are selected by the plant, but are not species specific. *Frontiers in Microbiology* **7**, doi:10.3389/fmicb.2016.00440 (2016).
