# Supplementary material for: Terrestrial-type nitrogen-fixing symbiosis between seagrass and a marine bacterium
Source: Nature. 2021 Nov 3;600(7887):105–9. doi: 10.1038/s41586-021-04063-4 (PMC8636270; doi:10.1038/s41586-021-04063-4)
Supplement: Supplementary file 2 — Reporting Summary [file 41586_2021_4063_MOESM2_ESM.pdf]

nature portfolio

Corresponding author(s): Wiebke Mohr

Last updated by author(s): Sep 2, 2021

Reporting Summary

Nature Portfolio wishes to improve the reproducibility of the work that we publish. This form provides structure for consistency and transparency in reporting. For further information on Nature Portfolio policies, see our Editorial Policies and the Editorial Policy Checklist.

Statistics

For all statistical analyses, confirm that the following items are present in the figure legend, table legend, main text, or Methods section.

n/a	Confirmed	
☐	☒	The exact sample size (*n*) for each experimental group/condition, given as a discrete number and unit of measurement
☐	☒	A statement on whether measurements were taken from distinct samples or whether the same sample was measured repeatedly
☐	☒	The statistical test(s) used AND whether they are one- or two-sided<br>*Only common tests should be described solely by name; describe more complex techniques in the Methods section.*
☒	☐	A description of all covariates tested
☐	☒	A description of any assumptions or corrections, such as tests of normality and adjustment for multiple comparisons
☐	☒	A full description of the statistical parameters including central tendency (e.g. means) or other basic estimates (e.g. regression coefficient) AND variation (e.g. standard deviation) or associated estimates of uncertainty (e.g. confidence intervals)
☒	☐	For null hypothesis testing, the test statistic (e.g. *F*, *t*, *r*) with confidence intervals, effect sizes, degrees of freedom and *P* value noted<br>*Give P values as exact values whenever suitable.*
☒	☐	For Bayesian analysis, information on the choice of priors and Markov chain Monte Carlo settings
☒	☐	For hierarchical and complex designs, identification of the appropriate level for tests and full reporting of outcomes
☒	☐	Estimates of effect sizes (e.g. Cohen's *d*, Pearson's *r*), indicating how they were calculated

Our web collection on statistics for biologists contains articles on many of the points above.

Software and code

Policy information about availability of computer code

Data collection	Zeiss ZEN 3.2 blue edition, xT microscope control software v6.2.6. For the acquisition of raw mass spectrometric and raw sequencing data, instrument-supplied software was utilized.
Data analysis	Matlab 2018b (Mathworks), QIIME2 (and the following plugins: cutadapt v1.16, VSEARCH 2.14.1, Deblur 1.1.0, q2-feature-classifier 2020.2.0.dev0, alpha-group-significance, DEICODE v0.2.3, q2-emperor 2020.2.0.dev0, beta-group-significance), Songbird v1.0.0, Qurro v0.5.0., phyloseq v1.22.3, phyloFlash 3.0/3.3b3, bbmap v38.75, metaFlye version 2.7, SAMtools version 1.10, Pilon version 1.23, CheckM version 1.0.18, Prokka 1.13.3, BWA-0.7.17, minimap2-2.17, dbCAN meta server (incl. HMMER, DIAMOND and Hotpep), RAST annotation server, SINA aligner 1.2.11, ARB 6.1, Trimmomatic 0.32, SortMeRNA 2.1, Bowtie2 2.1.0, samtools 0.1.19, featureCounts 1.4.6, BRIG v0.95, look@NanoSims v2018, Microsoft Excel. Custom codes for processing of eddy correlation data and nanoSIMS data can be found at github under https://github.com/SoerenAhmerkamp/EddyCorrelation and https://github.com/SoerenAhmerkamp/NanoSIMS/.

For manuscripts utilizing custom algorithms or software that are central to the research but not yet described in published literature, software must be made available to editors and reviewers. We strongly encourage code deposition in a community repository (e.g. GitHub). See the Nature Portfolio guidelines for submitting code & software for further information.

Data

Policy information about availability of data

All manuscripts must include a data availability statement. This statement should provide the following information, where applicable:

- Accession codes, unique identifiers, or web links for publicly available datasets
- A description of any restrictions on data availability
- For clinical datasets or third party data, please ensure that the statement adheres to our policy

Raw reads of the 16S rRNA gene amplicon sequencing, the MAGs of C. neptuna and C. diazotrophica (DSM18577), and the mapped reads of the transcriptomes are available under Bioproject number PRJEB37438 at the European Nucleotide Archive (ENA). Sequences that were included in the phylogenetic tree are available in Supplementary File 1 (with accession numbers and references) and as a tree file (Supplementary File 2). The comparison of 34 genomes for presence/absence of specific genes and/or pathways is available in Supplementary File 3 including their accession numbers. The PhyloFlash results (as presented in Extended Data Fig. 2) are available in Supplementary File 4. Publicly available sequences used for phylogenetic tree construction and genome comparison can be found under their respective accession numbers at NCBI (https://www.ncbi.nlm.nih.gov/) or ENA (https://www.ebi.ac.uk/ena/browser/home). Ribosomal subunit databases used for taxonomic classification can be found at the SILVA rRNA database (https://www.arb-silva.de/). Source data are provided with this paper.

Field-specific reporting

Please select the one below that is the best fit for your research. If you are not sure, read the appropriate sections before making your selection.

☐ Life sciences ☐ Behavioural & social sciences ☒ Ecological, evolutionary & environmental sciences

For a reference copy of the document with all sections, see nature.com/documents/nr-reporting-summary-flat.pdf

Ecological, evolutionary & environmental sciences study design

All studies must disclose on these points even when the disclosure is negative.

Study description	We carried out several field sampling campaigns to Posidonia oceanica meadows in the Mediterranean Sea. Sampling and experiments were set up to i) measure in situ rates of primary production and O2 penetration into the sediment, ii) measure N2 fixation rates associated with the roots of the seagrass and the subsequent transfer of freshly fixed N, iii) to subsample these rate incubations to visualize N2-fixing microorganisms, and iv) to study their potential metabolism via sequencing of metagenomes and metatranscriptomes. This study was designed to obtain a mechanistic insight into the interactions of N2-fixing microorganisms with the P. oceanica plant.
Research sample	The seagrass Posidonia oceanica is one of the most prolific seagrasses, producing large amounts of biomass. Its growth in a nutrient-poor environment indicates that microbial N2 fixation is important for this ecosystem. Our prior work showed that N2 fixation was mostly associated with the roots of the seagrass. We therefore focused on studying the root-associated N2 fixation activity as well as the transfer of the newly fixed N to the leaves. Sediment, pore water and the overlying water column were also sampled to obtain environmental data relevant to our study. Fetovaia Bay (Elba, Italy) was chosen as a study site due to its pristine nature and the oligotrophic conditions representative of Posidonia meadows. Further, the study site is near a field station with sampling and laboratory infrastructure, which allowed repeated sampling and the completion of experiments.
Sampling strategy	The manuscript reports data from several field campaigns across different seasons and years. Sampling size was largely determined by the feasibility of experiments with triplicate incubated plants for each set of experiments and at least one experiment per sampling campaign. The three replicate plants for each experimental set is warranted by the various sampling campaigns across different seasons.
Data collection	Eddy correlation measurements were carried out in situ during 13-24 hour deployments. Diving staff of Hydra Marine Sciences, Hydra Field work and Soeren Ahmerkamp were present during the deployment and recovery of the instrumentation (high-frequency current meters combined with fast O2 microsensors; sediment microsensors). Water column and sediment O2 concentrations were taken automated during this deployment time. Plant and sediment samples were collected by staff of Hydra Marine Sciences and Hydra Field Work. Nutrient data (fluorometrically/photometrically) was collected either in the nearby laboratory on Elba Island, Italy, (collected by Nadine Lehnen, Hannah Marchant, Wiebke Mohr and/or technical support staff) or using an autoanalyzer at the Max Planck Institute Bremen (operated by technical support staff). Biomass and mass spectrometric data was collected using an elemental analyzer coupled to a continuous-flow isotope ratio mass spectrometer (equipped with an autosampler, operated by technical support staff), a nanoscale secondary ion mass spectrometer (operated by Sten Littmann and technical support staff), a gas chromatograph and a gas chromatograph coupled to an isotope ratio mass spectrometer (operated by technical support staff; Eawag). Sequencing data was collected at the Max Planck Genome Centre Cologne (individual sequencing platforms are detailed in the methods section).
Timing and spatial scale	Field campaigns took place in June 2014, May 2015, April 2016, August 2016, May 2017, September 2018, June and September 2019. Different seasons were chosen to observe changes in microbial community and processes with a change from conditions where nutrients are available to nutrient-deplete conditions. The same seagrass meadow was visited during these campaigns (Fetovaia Bay, Elba), and an additional meadow at a different island (Cala della Ruta, Pianosa) was visited once in May 2017.<br>Fetovaia Bay opens easterly/southeasterly to the Mediterranean Sea with a maximum North-South and West-East extent of ~700 m. Cala della Ruta Bay opens southerly to the Mediterranean and has a maximum North-South and West-East extent of about 400 and 700 m, respectively.

Data exclusions	We excluded the sequencing data of three plants from the microbial community analyses (16S rRNA gene amplicon sequencing) because they did not pass our initial quality assessment (described in methods). These three plants belonged the largest group of plants for one (non-N2-fixing) season (May).
Reproducibility	All experiments described in our study are individual environmental sampling campaigns at different times and/or different years. Within each set of experiments, triplicate incubations and multiple measurements within each triplicate (where applicable) were performed to assess variability. Variability within triplicates was substantial (see Fig. 1, 2 and Extended Data Fig. 3) reflecting biological differences between individual plants and plant pieces rather than errors in measurements. All attempts at replication were successful.
Randomization	The plants were randomly collected by divers in accessible spots and considering to minimize potential damage to the seagrass meadow. Incubation plants were randomly chosen from the pool of sampled plants making sure that each incubated plant had sufficient root, rhizome and leaf material for subsequent measurements.
Blinding	Blinding was not pertinent to our study because it did not include any animals and/or human research participants. In addition, blinding was not possible since many analyses were also carried out by the persons in charge of sampling and interpretation of the data was done by persons in charge of analyses.

Did the study involve field work? ☒ Yes ☐ No

Field work, collection and transport

Field conditions	Weather conditions were calm enough to allow SCUBA diving and sample collection. Water temperatures were 16 °C (April), 18 °C (May 2015 and 2017), 23 °C (June 2014), 24 °C (June 2019), 26 °C (August 2016), 25 °C (September 2018) and 22 °C (September 2019). Nutrient concentrations in the water column above the seagrass meadow and in porewaters of seagrass and neighboring sediments were measured during several campaigns and are detailed in Extended Data Table 1.
Location	Posidonia oceanica meadow and neighboring sediments in Fetovaia Bay, Elba Island (Italy; 42°43.804'N 10°09.422'E) and Posidonia oceanica meadow in Cala della Ruta Bay, Pianosa Island (Italy; 42°34.362'N 010°03.795'E). Sampled meadows and sediments are at water depth between 5-10 m.
Access & import/export	The Posidonia oceanica meadows were accessed using minimal-invasive zodiacs/small boats and SCUBA-diving/snorkling. Local, national and international laws were followed. Access to the protected waters of the Island of Pianosa was granted by the National Park Tuscan Archipelago, Portoferraio, Italy (permit no. 2930/2017).
Disturbance	Plants were carefully separated from the meadow by hand to minimize damage/disturbance to the ecosystem. In situ measurements were minimally invasive.

Reporting for specific materials, systems and methods

We require information from authors about some types of materials, experimental systems and methods used in many studies. Here, indicate whether each material, system or method listed is relevant to your study. If you are not sure if a list item applies to your research, read the appropriate section before selecting a response.

Materials & experimental systems

n/a	Involved in the study
☒	☐ Antibodies
☒	☐ Eukaryotic cell lines
☒	☐ Palaeontology and archaeology
☒	☐ Animals and other organisms
☒	☐ Human research participants
☒	☐ Clinical data
☒	☐ Dual use research of concern

Methods

n/a	Involved in the study
☒	☐ ChIP-seq
☒	☐ Flow cytometry
☒	☐ MRI-based neuroimaging
